# Neural sampling machine with stochastic synapse allows brain-like learning and inference

**DOI:** 10.1038/s41467-022-30305-8

**Published:** 2022-05-11

**Authors:** Sourav Dutta, Georgios Detorakis, Abhishek Khanna, Benjamin Grisafe, Emre Neftci, Suman Datta

**Affiliations:** 1 Department of Electrical Engineering, University of Notre Dame, Notre Dame, IN 46556 USA; 2 Department of Cognitive Sciences, University of California Irvine, Irvine, CA 92697 USA

**Keywords:** Electrical and electronic engineering, Electronic devices, Electronic devices, Nanoscale materials, Electronics, photonics and device physics

## Abstract

Many real-world mission-critical applications require continual online learning from noisy data and real-time decision making with a defined confidence level. Brain-inspired probabilistic models of neural network can explicitly handle the uncertainty in data and allow adaptive learning on the fly. However, their implementation in a compact, low-power hardware remains a challenge. In this work, we introduce a novel hardware fabric that can implement a new class of stochastic neural network called Neural Sampling Machine (NSM) by exploiting the stochasticity in the synaptic connections for approximate Bayesian inference. We experimentally demonstrate an in silico hybrid stochastic synapse by pairing a ferroelectric field-effect transistor (FeFET)-based analog weight cell with a two-terminal stochastic selector element. We show that the stochastic switching characteristic of the selector between the insulator and the metallic states resembles the multiplicative synaptic noise of the NSM. We perform network-level simulations to highlight the salient features offered by the stochastic NSM such as performing autonomous weight normalization for continual online learning and Bayesian inferencing. We show that the stochastic NSM can not only perform highly accurate image classification with 98.25% accuracy on standard MNIST dataset, but also estimate the uncertainty in prediction (measured in terms of the entropy of prediction) when the digits of the MNIST dataset are rotated. Building such a probabilistic hardware platform that can support neuroscience inspired models can enhance the learning and inference capability of the current artificial intelligence (AI).

## Abstract (web summary)

Neural sampling machines make use of noise to perform learning. Here, Dutta et al. present a hybrid stochastic synapse composed out of a ferroelectric transistor combined with a stochastic selector exhibiting multiplicative synaptic noise required for implementing a neural sample machine.

## Introduction

**Fig. 1:**
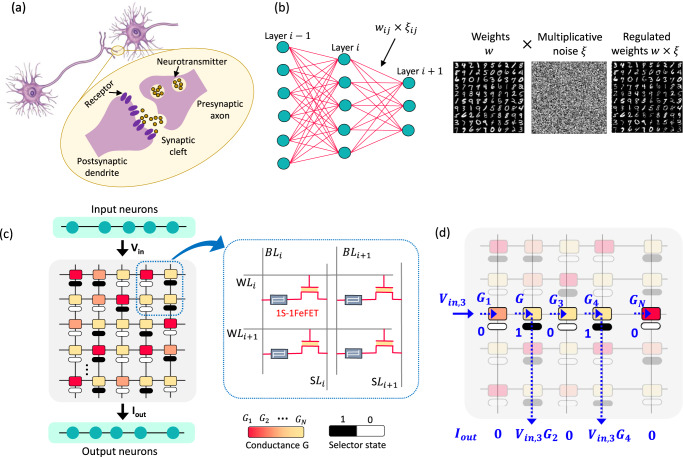
Overview of stochastic synapse. **a** Synaptic stochasticity occurring at the molecular level in biological neural networks. The presynaptic neuronal spike causes the release of neurotransmitters at the synaptic release site with a probability around 0.1. **b** Schematic of a Neural Sampling Machine (NSM) incorporating a Bernoulli or “blank-out” multiplicative noise in the synapse. This acts as a continuous DropConnect mask on the synaptic weights such that a subset of the weights is continuously forced to be zero. **c** Illustration of an NSM implemented in a hardware using crossbar array architecture implementing compute-in-memory. The analog weight cell implemented using eNVMs are placed at each cross-point and are augmented with a stochastic selector element. This allows selectively sampling or reading the synaptic weights *G*_*ij*_ with some degree of uncertainty, based on random binary variables *ξ*_*ij*_ generated for each of the synapse. **d** Illustration of a scenario where an input voltage *V*_*in,*3_ is applied to a row of the synaptic array with conductance states ${{{{{\boldsymbol{G}}}}}}=\{{G}_{1},{G}_{2},{G}_{3},{G}_{4},\ldots ,{G}_{N}\}$. Depending on the state of the selectors in the cross-points, an output weighted sum current ${{{{{{\boldsymbol{I}}}}}}}_{{{{{{\boldsymbol{out}}}}}}}=\{0,G_{2}{V}_{{in},3},0,{G}_{4}{V}_{{in},3},\ldots ,0\}$ is generated which is exactly same as multiplying the weight sum of ${w}_{{ij}}{z}_{j}$ with a multiplicative noise ${\xi }_{{ij}}$. WL word line, BL bit line, SL source line, *V*_in_ input voltage, *I*_out_ output current, *G* conductance.

Harnessing the intricate dynamics at the microscopic level in emerging materials and devices have unraveled new possibilities for brain-inspired computing such as building analog multi-bit synapses^1–10^ and bio-inspired neuronal circuits^10–12^. Such emerging materials and devices also exhibit inherent stochasticity at the atomic level which is often categorized as a nuisance for information processing. In contrast, variability is a prominent feature exhibited by biological neural networks at the molecular level are believed to contribute to the computational strategies of the brain^13^. Such variability has been reported in the recordings of biological neurons or as unreliability associated with the synaptic connections. Typically, a presynaptic neuronal spike causes the release of neurotransmitters at the synaptic release site as illustrated in Fig. 1a. Borst et. al.^14^ reported that the synaptic vesicle release in the brain can be extremely unreliable. The transmission rate can be as high as 50% and as low as 10% measured in vivo at a given synapse. Synaptic noise has the distinguishing feature of being multiplicative which plays a key role in learning and probabilistic inference dynamics. In this work, we propose a novel stochastic synapse that harnesses the inherent variability present in emerging devices and mimic the dynamics of a noisy biological synapses. This allows us to realize a novel neuromorphic hardware fabric that can support a recently proposed class of stochastic neural network called the Neural Sampling Machine (NSM)^15^.

While the functional role of this multiplicative stochasticity in the brain is still under debate, the biologically inspired stochasticity can be exploited in certain machine learning algorithms. In particular, NSMs build on the idea of introducing stochasticity at various levels in a neural network to allow—(1) escaping local minima during learning and inference^16^, (2) regularization in neural networks^17,18^, (3) approximate Bayesian inference with Monte-Carlo sampling^19,20^ and (4) energy efficient communication and computation^21,22^. NSM draws inspiration from regularization techniques such as Dropout^17^ or DropConnect^18^ that randomly drop a subset of neural activation or weights in the neural network during the forward pass of training. Contrary to DropConnect where stochasticity is switched off during inference, the synaptic stochasticity is always present in an NSM. This “always-on” stochasticity confers probabilistic inference capabilities to the network^20^ and is consistent with the idea of continual learning and lifelong learning machines while improving energy efficiency^21,22^. Neural networks equipped with “always-on“ stochasticity have been shown to match or surpass the performance of contemporary machine learning algorithms. Together with multiplicative noise incorporated in stochastic synapses, this new class of NSM provides an important pathway toward realizing probabilistic inference^23^ and active learning^24,25^,

In this work, we propose a hardware implementation of NSM using hybrid stochastic synapses consisting of an embedded non-volatile memory (eNVM) in series with a two-terminal stochastic selector element. We experimentally demonstrate in silico such a hybrid stochastic synapse by pairing a doped HfO_2_ FeFET-based analog weight cell with a two-terminal Ag/HfO_2_ stochastic selector. Such hybrid synapses can be integrated within the prevailing crossbar array architecture for CIM that provides a promising energy-efficiency pathway for building neuromorphic hardware by reducing data-movement^26^. We exploit the inherent stochastic switching of the selector element between the insulator and the metallic state to perform Bernoulli sampling of the conductance states of the FeFET both during learning and inference. A remarkable feature of the multiplicative noise dynamics is a self-normalizing effect that performs automatic weight normalization and prevention of internal covariate shift in an online fashion. Furthermore, the “always-on” stochasticity of the NSM during the inference mode allows performing Bayesian inferencing.

## Theoretical model of NSM

NSM are stochastic neural networks that exploit neuronal and/or synaptic noise to perform learning and inference^15^. A schematic illustration is shown in Fig. 1b comprising synaptic stochasticity that injects a multiplicative Bernoulli or “*blank-out*” noise in the model. Such a noise can be incorporated in the model as a continuous DropConnect^18^ mask on the synaptic weights such that a subset of the weights is continuously forced to be zero as shown in Fig. 1b. Next, we lay down a theoretical description of the NSM.

We use binary threshold neurons with the following activation function:

1 $${z}_{i}={{{{{{\rm{sgn}}}}}}}\left({u}_{i}\right)=\left\{\begin{array}{c}-1,{{{{{{\rm{if}}}}}}}\,{u}_{i} \, < \, 0\\ 1,{{{{{{\rm{if}}}}}}}\,{u}_{i}\ge 0\end{array}\right.$$

where *u*_*i*_ is the pre-activation of neuron *i* and is given by:

2 $${u}_{i}=\mathop{\sum }\limits_{j=1}^{N}(\xi _{ij}+a_{i})w_{ij}\,{z}_{j}+b_{i}$$

where *w*_*ij*_ represents the weight of the synaptic connection between neurons *i* and *j* and *ξ*_*ij*_ is the multiplicative Bernoulli noise modeled using an independent and identically distributed (iid) random variable with parameter *p* such that ${\xi }_{{ij}} \sim {{{{{{\rm{Bernoulli}}}}}}}\left(p\right)\in [{{{{\mathrm{0,1}}}}}]$. *b*_*i*_ is a bias term applied per neuron *i*. An additional term *a*_*i*_ is added per neuron *i* to counter the scaling factor issue due to multiplicative noise^27^. It can be further shown that for such binary threshold neurons, the probability of a neuron firing is given by:

3 $$P\left({z}_{i}=1|{{{{{\boldsymbol{z}}}}}}\right)=\frac{1}{2}\left[1+{{{{{\rm{erf}}}}}}\left(\frac{{\mathbb{E}}\left({u}_{i}|{{{{{\boldsymbol{z}}}}}}\right)}{\sqrt{2{{{{{{\rm{Var}}}}}}}\left({u}_{i}|{{{{{\boldsymbol{z}}}}}}\right)}}\right)\right]$$

where ${\mathbb{E}}({u}_{i})$ and Var(*u*_*i*_) are the expectation and variance of *u*_*i*_. For Bernoulli type noise, the probability of neuron firing becomes^27^:

4 $$P({z}_{i}=1|{{{{{\boldsymbol{z}}}}}}) =\frac{1}{2}\left[1+{{{{{\rm{erf}}}}}}\left(\frac{\left(p+{a}_{i}\right)\mathop{\sum}\limits_{j}{w}_{ij}{z}_{j}}{\sqrt{2p(1-p)\mathop{\sum}\limits_{j}{w}_{ij}^{2}}}\right)\right]\\ =\frac{1}{2}\left[1+{{{{{\rm{erf}}}}}}\left(\frac{(p+{a}_{i})\mathop{\sum}\limits_{j}{w}_{ij}{z}_{j}}{\sqrt{2p(1-p)}\|{{{{{\boldsymbol{w}}}}}}_{{{{{{\boldsymbol{i}}}}}}}\|}\right)\right]\\ =\frac{1}{2}\left[1+{{{{{\rm{erf}}}}}}\left({\beta }_{i}\frac{\mathop{\sum}\limits_{j}{w}_{{ij}}{z}_{j}}{\|{{{{{{\boldsymbol{w}}}}}}}_{{{{{{\boldsymbol{i}}}}}}}\|}\right)\right]=\frac{1}{2}\left[1+{{{{{\rm{erf}}}}}}\left({{{{{{\boldsymbol{v}}}}}}}_{{{{{{\boldsymbol{i}}}}}}}{{{{{\boldsymbol{.}}}}}}{{{{{\boldsymbol{z}}}}}}\right)\right]$$

with $\beta =\frac{p+{a}_{i}}{\sqrt{2p(1-p)}}$ capturing the noise in the model and ${v}_{i}={\beta }_{i}\frac{{{{{{{\boldsymbol{w}}}}}}}_{{{{{{\boldsymbol{i}}}}}}}}{{||}{{{{{{\boldsymbol{w}}}}}}}_{{{{{{\boldsymbol{i}}}}}}}{{{{{\boldsymbol{||}}}}}}}$. Here, ${||}{{{{{\boldsymbol{\cdot }}}}}}{{{{{\boldsymbol{||}}}}}}$denotes the L2 norm of the weights of neuron *i*. Note that the notion behind weight normalization is to re-parameterize the weight vector using ${v}_{i}={\beta }_{i}\frac{{{{{{{\boldsymbol{w}}}}}}}_{{{{{{\boldsymbol{i}}}}}}}}{{||}{{{{{{\boldsymbol{w}}}}}}}_{{{{{{\boldsymbol{i}}}}}}}{{{{{\boldsymbol{||}}}}}}}$^28^ which is exactly the same as that obtained in NSM due to the inherent stochastic noise in the synapses. Thus, NSM inherently introduces the salient self-normalizing feature and performs weight normalization in the same sense as^28^. One important feature of the NSM is that since this weight normalization is an inherent feature of the model, NSM offers the features equivalent to batch normalization in an online fashion. Additionally, by decoupling the magnitude and the direction of the weight vector, a potential speedup in convergence is obtained^27^.

## Implementing NSM using emerging devices operating in stochastic switching regime

Recent years have seen extensive research on building dedicated hardware for accelerating DNNs using CIM approach. The core computing kernel consists of a crossbar array with perpendicular rows and columns with eNVMs placed at each cross-point as shown in Fig. 1c. The weights in the DNN are mapped to the conductance states of the eNVM. The crossbar array performs row-wise weight update and column-wise summation operations in a parallel fashion as follows: the input (or read) voltages ***V***_***in***_ from the input neuron layer are applied to all the rows and are multiplied by the conductance of the eNVM at each cross-point ***G*** to create a weighted sum current in each column ${{{{{{\boldsymbol{I}}}}}}}_{{{{{{\boldsymbol{out}}}}}}}=\sum {{{{{\boldsymbol{G}}}}}}{{{{{{\boldsymbol{V}}}}}}}_{{{{{{\boldsymbol{in}}}}}}}$. The output neuron layer placed at the end of the column converts these analog currents into digital neuronal outputs.

Implementing an NSM with the same existing hardware architecture requires selectively sampling or reading the synaptic weights *G*_*ij*_ with some degree of uncertainty, based on random binary variables *ξ*_*ij*_ generated for each of the synapse. We show that this can be easily realized by pairing the eNVM such as FeFET in series with a two-terminal stochastic selector element at each cross-point as shown illustratively in Fig. 1c. We choose a selector device such that it operates as a switch, stochastically switching between an ON state (representing ${\xi }_{{ij}}=1$) and an OFF state (${\xi }_{{ij}}=0$). The detailed description of such a selector is mentioned later. Figure 1d shows a scenario where an input voltage *V*_*in,3*_ is applied to the third row of the synaptic array while the conductance of the synapses are set to ${{{{{\boldsymbol{G}}}}}}=\{{G}_{1},{G}_{2},{G}_{3},{G}_{4},\ldots ,{G}_{N}\}$. Depending on the state of the selectors in the cross-points, an output weighted sum current ${{{{{{\boldsymbol{I}}}}}}}_{{{{{{\boldsymbol{out}}}}}}}=\{0,{G}_{2}{V}_{{in},3},0,{G}_{4}{V}_{{in},3}, \ldots , 0\}$ is generated. This is the same as multiplying the weight sum of ${w}_{{ij}}{z}_{j}$ with a multiplicative noise *ξ*_*ij*_ as described in Eq. (2).

## Building blocks for stochastic synapse: FeFET-based analog weight cell

**Fig. 2:**
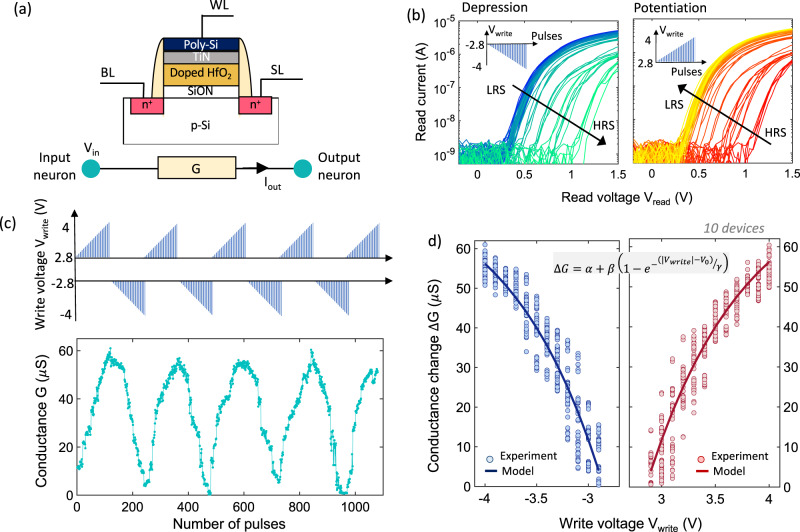
FeFET-based analog synapse. **a** Schematic of a stand-alone FeFET-based analog synapse. The channel conductance can be modulated by applying write pulses ±*V*_write_ to the gate of the FeFET while reading out the conductance state is achieved by applying a small read voltage *V*_read_ to the gate terminal. **b** Experimentally measured conductance modulation in a 500 nm × 500 nm high-K metal gate FeFET fabricated at 28 nm technology node. An amplitude modulation scheme is used where positive and negative write voltage pulses *V*_write_ of increasing amplitude from 2.8 V to 4 V and pulse widths of 1 μs are applied to modulate the conductance of the FeFET. **c** Measured continuous change in the conductance state of the FeFET upon applying multiple potentiation and depression pulses of varying amplitude. **d** The FeFET-based analog weight cell is modeled in the NSM by fitting the conductance update scheme for both potentiation and depression with the closed-form expression as shown in the figure. WL word line, BL bit line, SL source line, *V*_in_ input voltage, *I*_out_ output current, *V*_write_ write voltage, *G* conductance, LRS low resistance state, HRS high resistance state.

The idea of voltage-dependent partial polarization switching in ferroelectric Hf_x_Zr_1-x_O_2_ can be leveraged to implement a non-volatile FeFET-based analog synapse. The FeFET-based synapse can be integrated into a pseudo-crossbar array following different memory array topologies such as NOR array, AND array etc.^29,30^. The illustration of the pseudo-crossbar array shown in Fig. 1c is similar to an AND memory array architecture. Figure 2a shows the schematic of a FeFET-based analog synapse (without any additional stochastic selector element) where the gate, drain and source of the FeFET are connected to the word-line (WL), bit-line (BL) and source-line (SL), respectively. The channel conductance *G* of the FeFET can be gradually modulated by applying write voltage pulses to the gate of the FeFET. During the write operation, a write voltage ± *V*_write_ is applied to the gate of the FeFET through the WL. The source and drain of the FeFET are kept grounded by applying 0 V to the BL and SL. During the read operation, a read voltage *V*_read_ = 1 V is applied to the gate through the WL while *V*_in_ is applied to the drain through BL and the SL is grounded. Note that the applied *V*_in_ must be within the range of the threshold voltages of the selector devices in order to implement the stochastic synapse as explained later. Thus, during the readout phase, the output (drain) current from the FeFET becomes ${I}_{{{{{{{\rm{out}}}}}}}}=G{V}_{{{{{{{\rm{in}}}}}}}}$.

Note that such an array topology is suitable for row-wise weight update and column-wise summation^6,10,31^. For example, in the AND array topology shown in Fig. 1c, the BL and SL run parallel while the WL is orthogonal. For configuring such an AND array, two write inhibition schemes can be used: *V*_write_/2 and *V*_write_/3^29,30^. For the *V*/2 scheme, the target FeFET to be programmed or erased experiences the full write voltage *V*_write_ across it. On the other hand, the half-selected cells experience a write disturb voltage of *V*_write_/2, while the unselected cells do not experience any write disturb. For the *V*_write_/3 scheme, the half-selected and unselected cells experience a write disturb voltage of *V*_write_/3 and −*V*_write_/3, respectively. Thus, by applying appropriate voltages in the BLs and SLs, we can have for row-wise parallel weight update. However, note that the program and erase operation needs to be done in two separate phases.

Figure 2b shows the experimentally measured conductance modulation in a 500 nm × 500 nm high-K metal gate FeFET fabricated at 28 nm technology node^32^. For online learning on crossbar arrays, typically potentiation and depression pulse schemes with identical pulse amplitudes and widths are preferred. Nonetheless for a proof-of-concept, we used an amplitude modulation scheme where write voltage pulses *V*_write_ of increasing amplitude from 2.8 V to 4 V and pulse widths of 1 μs are applied to modulate the conductance of the FeFET. Applying progressively increasing negative pulses causes the FeFET to transition from the initial low resistance state (LRS) with lower threshold voltage (*V*_*T*_) to high resistance state (HRS) as shown by the current-voltage characteristics in Fig. 2b. Similarly, applying progressively increasing positive pulses causes a change in the conductance from HRS to LRS. Figure 2c shows a continuous change in the conductance state of the FeFET upon applying multiple potentiation and depression pulses of varying amplitude and constant pulse width of 1 μs. The cycle-to-cycle variation in the measured conductance states observed in Fig. 2c arises due to the inherent stochastic switching dynamics of the individual ferroelectric domains^33^. Such inherent stochasticity also results in a device-to-device variation of the conductance states. To incorporate such variability, we measured the conductance modulation both for potentiation and depression across ten devices as shown in Fig. 2d. We incorporate the model of FeFET-based analog weight cell in the NSM by fitting the conductance update scheme for both potentiation and depression with the closed-form expression $\Delta G=\alpha +\beta (1-{e}^{-\left(|{V}_{{{{{{{\rm{write}}}}}}}}|-{V}_{0}\right)/\gamma })$ where *α, β, γ* and ${V}_{0}$ are the fitting parameters.

## Building blocks for stochastic synapse: Ag/HfO_2_ stochastic selector

**Fig. 3:**
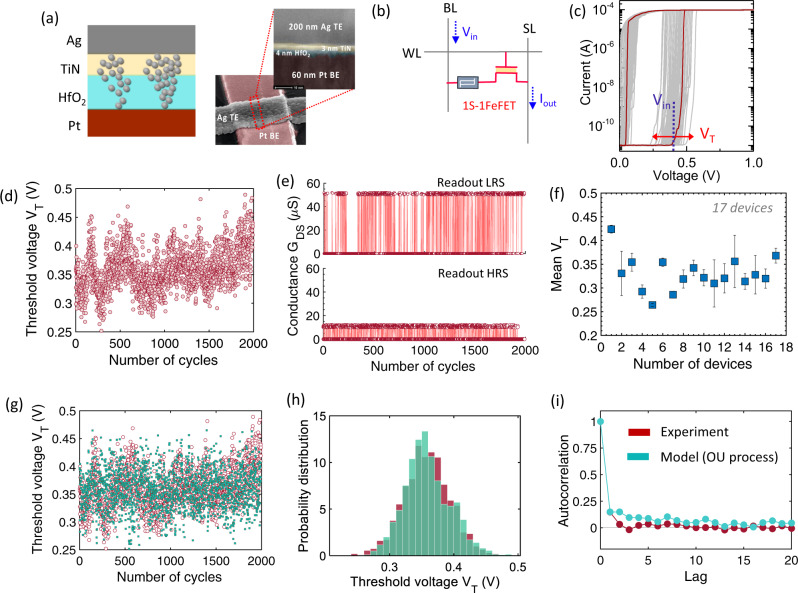
Introducing multiplicative noise through stochastic selector. **a** Schematic and TEM of a fabricated stack of [Ag/TiN/HfO_2_/Pt] with 3 nm TiN and 4 nm HfO_2_. **b** A stochastic synapse is realized by augmenting this stochastic selector in series with the FeFET-based analog weight cell. **c** Measured current-voltage characteristics showing abrupt electronic transition from insulating state to metallic state due to the formation of a continuous filament of Ag+ atoms bridge the top and bottom electrodes. A wide window of variation in the threshold voltage *V*_*T*_ that triggers the spontaneous formation of the Ag^+^ filament is observed. The stochasticity can be exploited by applying the input voltage *V*_in_ within the variation window of the *V*_*T*_. **d** Measured threshold voltage *V*_*T*_ over multiple cycles. **e** Stochastically reading an LRS and an HRS of the FeFET through the stochastic selector. **f** Measured device-to-device variation across 17 selector devices. Error bar denotes standard deviation across the mean. **g**–**i** The stochasticity switching of the selector device is modeled using an Onrstein-Uhlenbeck (OU) Process. The model shows excellent agreement with the experimental data. WL word line, BL bit line, SL source line, *V*_in_ input voltage, *I*_out_ output current, LRS low resistance state, HRS high resistance state.

Next, we describe the characteristics of our stochastic selector device. Figure 3a shows a schematic and a transmission electron microscopy of a fabricated stack of [Ag/TiN/HfO_2_/Pt] with 3 nm TiN and 4 nm HfO_2_. A stochastic synapse is realized by augmenting this stochastic selector in series with the FeFET-based analog weight cell as shown in Fig. 3b. The [Ag/TiN/HfO2/Pt] metal ion threshold switch device, from here on referred to as the Ag/ HfO_2_ selector device, operates based on the principle of metal ion migration through a metal oxide medium similar to conducting bridge RAM. Starting from an initial OFF state, under an applied external bias, Ag atoms ionize and respond to the electric field migrating via interstitial hopping from top electrode to bottom electrode until a continuous filament of Ag+ atoms bridge the top and bottom electrodes. This is accompanied by several orders of magnitude change in conductivity as the device turns ON. As the field is reduced, the inclination for Ag atoms to form clusters with other Ag atoms, rather than linear chains of atoms in contact with Pt allows for the spontaneous rupture of the atomic filament, turning OFF the device^34^. The role of TiN in the stack is to limit the initial migration of Ag during the electroforming sweep, such that device reliability is enhanced^35^. We perform DC current-voltage (IV) characterization of the selector device as shown in Fig. 3c. It is seen that upon repeated DC cycling, there is considerable variation in the threshold voltage *V*_*T*_ that triggers the spontaneous formation of the Ag^+^ filament through HfO_2_ insulating matrix. To precisely capture the threshold switching voltage and hold voltage of the selector device, we apply long pulses of 10 ms rise and fall time and 10 ms pulse width. Note that ultra-fast switching speed of the Ag/HfO_2_ selector has been previously reported to be around 28 ns^35^. With shorter read pulses (<50 ns), the required trigger voltage will increase. However, the stochastic nature of the selector will still be retained. Figure 3d shows the cycle-to-cycle variation in *V*_*T*_, measured across 2000 cycles.

The stochastic switching can be exploited by applying an input voltage *V*_in_ within the variation window of the *V*_*T*_ as shown in Fig. 3c. This would allow stochastic sampling of the conductance state of the FeFET in series. Figure 3e shows two examples of stochastically reading an LRS and an HRS of the FeFET through the stochastic selector. We additionally performed switching measurements on 17 selector devices to capture the device-to-device variation as shown in Fig. 3f. Overall, this validates the proposed idea of using such a hybrid structure as a truly stochastic synapse for implementing NSM on the hardware.

The stochastic switching of the selector device is incorporated in the NSM by modeling it as an Onrstein-Uhlenbeck (OU) Process. The OU process is a stochastic process (similar to diffusion), which was introduced as a generalized Brownian motion model (see Methods section for details). Using this modeling framework, the dynamics of the *V*_T_ can be described as:

5 $$d{V}_{T}=\theta \left(\mu -{V}_{T}\right){dt}+\sigma {dW}$$

where *W* is the Wiener process, *θ* describes the magnitude of the mean-reverting force toward the mean *μ*. *σ* captures the diverting variance. We calibrated the parameters of Eq. (5) using experimentally measured variation in the threshold voltage for all the 17 selector devices. Details of the OU calibration is included in the Methods section. The calibrated OU process shows excellent agreement with our experimental results as shown in Fig. 3g–i in terms of the cycle-to-cycle variation of *V*_*T*_, overall distribution of *V*_*T*_ and autocorrelation.

## Hardware NSM and image classification task

**Fig. 4:**
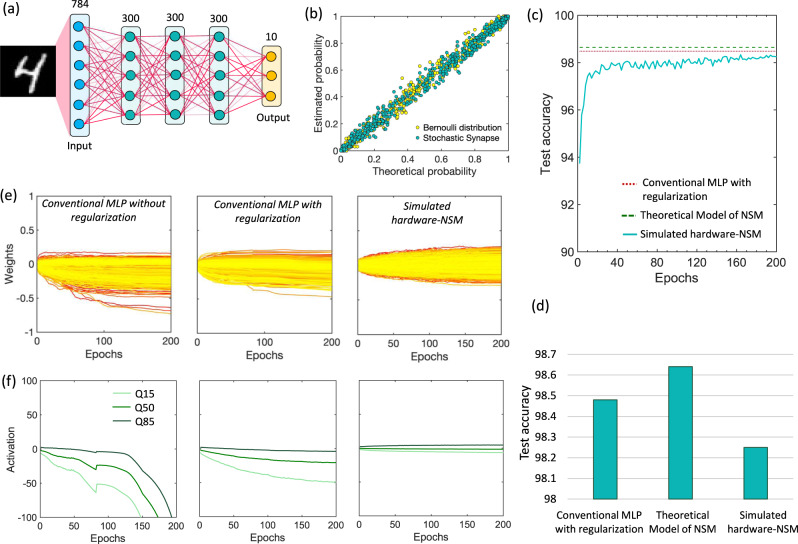
Hardware NSM performing image classification and exhibiting self-normalization. **a** Network architecture of the NSM consisting of an input layer, three hidden fully connected layers and an output layer. **b** Exact match witnessed between the measured switching probability of the stochastic selector device and theoretically predicted probability for a Bernoulli distribution, highlighting that our stochastic selector device can inject Bernoulli multiplicative noise. **c** Evolution of the test accuracy for the simulated hardware-NSM using the FeFET-based analog weight cell and the stochastic selector as a function of the epochs. **d** Comparison of the performance of the simulated hardware-NSM with a deterministic feedforward multilayer perceptron (MLP) and the theoretical NSM model with full precession synaptic weights and a Bernoulli multiplicative noise for the stochastic synapses. **e** Evolution of the weights of the third layer during learning for three different networks- an MLP without any regularization, an MLP with additional regularization added and the simulated hardware-NSM. **f** Evolution of the 15th, 50th and 85th percentiles of the input distributions to the last hidden layer during training for all the three networks. Overall, NSM exhibits a tighter distribution of the weights and activation concentrated around its mean, highlighting the inherent self-normalizing feature. MLP multilayer perceptron, NSM neural sampling machine, Q quantile.

We test the performance of our hardware NSM incorporating FeFET-based analog weight cell and stochastic selector as the hybrid stochastic synapse on image classification task using the MNIST handwritten digit dataset as an example. Figure 4a shows the network architecture consisting of an input layer with 784 neurons, three fully connected hidden layers with 300 neurons and a softmax output layer of 10 neurons for 10-way classification. For comparison, we chose three networks with the same architecture—(1) deterministic feedforward multilayer perceptron (MLP), (2) theoretical NSM model with full precession synaptic weights and a Bernoulli multiplicative noise for the stochastic synapses and (3) simulated hardware-NSM using the FeFET-based analog weight cell and the stochastic selector. The hardware NSM is trained using backpropagation and a softmax layer with cross-entropy loss and minibatch size of 100. While training of the hardware NSM, during the backward pass, the weight update is applied using the derivative of Eq. (4) and the closed-form equation in Fig. 2d. Like Dropout and Dropconnect schemes, the proposed NSM also uses a stochastic blank-out mask in the learning phase. This allows stochastically accessing the weights for the backward pass during the learning phase. However, in contrast to the Dropout or Dropconnect, the weights in an NSM are also accessed stochastically during the inference phase, leading to the concept of Monte-Carlo Dropout or "Always-on Dropout". We implement this by calculating the *V*_*T*_ of each selector device in the cross-points in every iteration using the OU process described by Eq. (5) and constructing a Boolean matrix *Ξ* such that if ${V}_{T}\ge {V}_{T,{{{{{{\rm{mean}}}}}}}}$, ${\xi }_{{ij}}=1$, else ${\xi }_{{ij}}=0$. Subsequently, we evaluate Eqs. (1) and (2).

The exact nature of the multiplicative noise injected by the stochastic selector is understood by comparing the measured switching probability with the theoretically predicted probability of switching for a Bernoulli process. Figure 4b shows an exact match between the measured and theoretically predicted probability, highlighting that our stochastic selector device can inject Bernoulli multiplicative noise. Figure 4c, d shows the performance of the hardware NSM in terms of the test accuracy and comparison with the theoretical NSM model and conventional MLP network. It is seen that the theoretical model outperforms the conventional MLP network as highlighted in ref. ^27^. The simulated hardware-NSM shows comparable test accuracy with the conventional MLP, the performance mainly limited by the dynamic range and non-idealities of the FeFET-based synaptic weight cell such as cycle-to-cycle and device-to-device variations, non-linearity and asymmetric change in conductance (potentiation and depression) as seen in Fig. 2c, d.

## Inherent weight normalization and robustness to weight fluctuations

As explained earlier, NSM allows decoupling the weight matrix as ${v}_{i}={\beta }_{i}\frac{{{{{{{\boldsymbol{w}}}}}}}_{{{{{{\boldsymbol{i}}}}}}}}{{||}{{{{{{\boldsymbol{w}}}}}}}_{{{{{{\boldsymbol{i}}}}}}}{{{{{\boldsymbol{||}}}}}}}$ which provides several advantages. Firstly, an inherent weight normalization can be effectively achieved without resorting to any batch normalization technique by performing gradient descent (calculating derivatives) with respect to the variables *β* in addition to the weights ***w*** as^27^:

6 $$\frac{\partial {{{{{\mathscr{L}}}}}}}{\partial {\beta }_{i}}=\frac{\mathop{\sum}\limits_{j}{w}_{{ij}}{\partial }_{{v}_{{ij}}}{{{{{\mathscr{L}}}}}}}{||{{{{{{\boldsymbol{w}}}}}}}_{{{{{{\boldsymbol{i}}}}}}}|{{{{{\boldsymbol{|}}}}}}}$$

7 $$\frac{\partial {{{{{\mathscr{L}}}}}}}{\partial {w}_{{ij}}}=\frac{{\beta }_{i}}{||{{{{{{\boldsymbol{w}}}}}}}_{{{{{{\boldsymbol{i}}}}}}}|{{{{{\boldsymbol{|}}}}}}}\frac{\partial {{{{{\mathscr{L}}}}}}}{\partial {v}_{{ij}}}-\frac{{\beta }_{i}}{{||{{{{{{\boldsymbol{w}}}}}}}_{{{{{{\boldsymbol{i}}}}}}}|{{{{{\boldsymbol{|}}}}}}}^{2}}{{{{{{\boldsymbol{w}}}}}}}_{{{{{{\boldsymbol{i}}}}}}}\frac{\partial {{{{{\mathscr{L}}}}}}}{\partial {\beta }_{i}}$$

This allows the distribution of the weights in the NSM to remain more stable than a conventional MLP without any additional weight regularization applied. Figure 4e shows the evolution of the weights of the third layer during learning for three cases—(1) an MLP without any regularization, (2) MLP with additional regularization added and (3) hardware NSM. It is seen that the distribution of NSM weights is narrower and remains concentrated around its mean (low variance). On the other hand, the variance of the weight distribution is larger for the MLP network without weight regularization. While we only show the evolution of the weights for the third layer during learning as a representative example, we expect similar behavior for the first and second layers. However, the effect might be smaller compared to the third layer.

## Mitigation of internal covariate shift

The internal covariate shift is defined as the change in the distribution of network’s activations due to a change in network’s parameters during training. In a deep neural network, the output of a previous layers acts as the input for the next layer. As such, a large change in the parameters of a particular layer can highly impact the distribution of the input into the next layer. These large shifts in the input distribution, a.k.a., the internal covariate shift, becomes problematic as the number of layers in the neural network increases. Recently, batch normalization has been proposed as an effective way to mitigate this problem^36^. Similar to batch normalization, the proposed NSM also exhibits a self-normalizing feature that prevents the internal covariate shift. To highlight this, we compare the 15th, 50th and 85th percentiles of the input distributions to the last hidden layer during training for all the three networks as shown in Fig. 4f. The internal covariate shift is clearly visible in the conventional MLP without any normalization incorporated as the input distributions change significantly during the learning. In contrast, the evolution of the input distribution in the hardware NSM is remains stable, suggesting that NSMs prevents internal covariate shift through the self-normalizing effect that inherently performs weight normalization as shown in Fig. 4e.

## Bayesian inferencing and capturing uncertainty in data

**Fig. 5:**
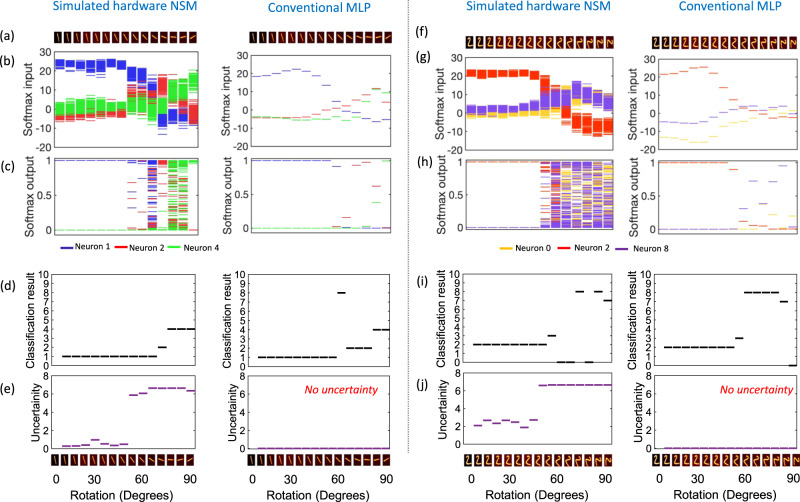
Bayesian inferencing and uncertainty in data comparison between simulated hardware-NSM and a conventional MLP network. **a**, **f** Continuously rotated images of the digits 1 and 2 from the MNIST dataset, used for performing Bayesian inferencing. We perform 100 stochastic forward passes during the inference mode for each rotated image of digits 1 and 2 and record the distribution of the (**b**, **g**) softmax input and (**c**, **h**) softmax output for few representative output neurons. **d**, **i** Classification result produced by the NSM for each rotated image. **e**, **j** The uncertainty of the NSM associated with the prediction, calculated in terms of the entropy *H* = −Σp*log$(p)$, where *p* is the probability distribution of the prediction. When the NSM makes a correct prediction (classifying image 1 and 2 as belonging to class 1 and 2, respectively), the uncertainty measured in terms of the entropy remains 0. However, in the case of wrong predictions, the uncertainty associated with the prediction becomes large. MLP multilayer perceptron, NSM neural sampling machine.

Next, we showcase the ability of our simulated hardware-NSM to perform Bayesian inferencing and produce classification confidence. For this, we train our hardware NSM on the standard MNIST dataset. During inference, we evaluate the ability to classify rotated images of digits from the MNIST dataset. Figure 5a, f shows digits 1 and 2 from the MNIST dataset, each rotated continuously by 6^0^. For each of the rotated images, we perform 100 stochastic forward passes and record the softmax input (output of the last fully connected hidden layer in Fig. 4a) as well the softmax output. We highlight the response of three representative neurons—1, 2 and 4 out of all the 10 neurons that show the highest activity. It is seen that when the softmax input of a particular neuron is larger than all the other neurons, the NSM will predict the class corresponding to that neuron. For example, in Fig. 5b–d, for the first seven images, the softmax input for neuron 1 is largest. Consequently, the softmax output for neuron 1 remains close to 1 and the NSM predicts the images as belonging to class 1. However, as the images are rotated more, it is seen that even though the softmax output can be arbitrarily high for neuron 2 or 4 predicting that the image belongs to the class 2 or 4, respectively, the uncertainty in the softmax output is high (output covering the entire range from 0 to 1). This signifies that the NSM can account for the uncertainty in the prediction. We quantify the uncertainty of the NSM by looking at the entropy of the prediction, defined as $H=-\sum p* {\log }\left(p\right)$, where *p* is the probability distribution of the prediction. As shown in Fig. 5d, e, when the NSM makes a correct prediction (classifying image 1 as belonging to class 1), the uncertainty measured in terms of the entropy remains 0. However, in the case of wrong predictions (classifying rotated image of 1 as belonging to class 2 or 4), the entropy associated with the prediction becomes large. This is in contrast to the results obtained from a conventional MLP network where the network cannot account for any uncertainty in the prediction and the entropy remains zero as shown in Fig. 5. Similar results are highlighted when presenting the NSM with rotated images of digit 2 as shown in Fig. 5f–j.

## Discussion

Stochasticity works a powerful mechanism in introducing many computational features of a deep neural network such regularization and Monte-Carlo sampling. This work builds upon the inherent weight normalization feature exhibited by a stochastic neural network, specifically the NSM. Such normalization acts as a powerful feature in most modern deep neural networks^28,36,37^, mitigating internal covariate shift and providing an alternative mechanism for divisive normalization in bio-inspired neural networks^38^. The proposed theoretical NSM model provides several advantages: (1) it is an online alternative for otherwise used batch normalization and dropout techniques, (2) it can mitigate saturation at the boundaries of fixed range weight representations, and (3) it provides robustness against spurious fluctuations affecting the rows of the weight matrix.

We demonstrate that the required stochastic nature of the theoretical NSM model can be realized in emerging stochastic devices. This allows seamless implementation of NSM on a hardware using the compute-in-memory architecture. We demonstrate the capability of our proposed hardware NSM to perform image recognition task on standard MNIST dataset with high accuracy (98.25%) comparable to state-of-the-art deterministic neural network. We also showcase the ability of our hardware NSM to perform probabilistic inferencing and quantify the uncertainty in data. Note that while this work focuses on using FeFET as the analog weight cell and Ag/HfO_2_ as the stochastic selector, a hardware NSM can also be realized using other emerging devices. For example, one can utilize emerging memory candidates such as PCM and RRAM instead of FeFET as the analog weight cell can.

For the stochastic selector, other candidates can be explored including ovonic threshold switch^39^, mixed ionic electronic conductor^40^, and insulator metal transition (IMT) oxides^41^ such as vanadium dioxide (VO_2_)^42,43^ and niobium oxide (NbO_x_)^44,45^. Note that while the endurance cycling of Ag/HfO_2_ based stochastic selector (>10^8^ cycles^35,46,47^) is sufficient for inference application. However, for on-chip training we can resort to insulator-to-metal phase transition (IMT)-based selectors such as vanadium dioxide (VO_2_)^48^ and niobium oxide (NbO_x_)^44^ that exhibits higher endurance cycling exceeding 10^9^.

The NSM hardware proposed in this work is primarily focused on an efficient and local implementation of the inference phase. An efficient and local implementation of the learning dynamics on-chip provides few additional challenges as the gradient backpropagation through the NSM requires backpropagating the errors through the network. This presents primarily three challenges: (a) bidirectional and symmetric connections, (b) multiplying errors by derivative of the activation functions as given in Eq. (4), and (c) computation of Eqs. (6) and (7). Challenges (a) and (b) are common to many other contemporary architectures for local learning on crossbar arrays. Challenge (a) can be sidestepped by using approximations of the gradient backpropagation such as feedback alignment or by using local loss functions (e.g., contrastive losses, or greedy layer-wise classifiers). The computation of the Gaussian activation Eq. (4) can be avoided by using “straight-through estimators”^49^ where the derivative function is replaced by 1, or using surrogate gradient functions without compromising the accuracy. Challenge (c) is however unique to the proposed NSM model. We speculate that it would require us to read the value of every afferent synaptic weight of a neuron to compute the norm. Furthermore, we speculate that Eqs. (6) and (7) could be computed approximately and more efficiently by ensuring that the norm of the weight (${||}{{{{{{\boldsymbol{w}}}}}}}_{{{{{{\boldsymbol{i}}}}}}}{{{{{\boldsymbol{||}}}}}}$) remains constant. However, further details on these approximations are beyond the scope of this work.

## Methods

### Fabrication of Ag/HfO_2_ stochastic selector

Ag/TiN/HfO_2_/Pt devices are fabricated on 250 nm SiO_2_/Si substrates. Bottom electrodes are patterned with e-beam lithography and 15 nm/60 nm Ti/Pt deposited via e-beam evaporation. A 4 nm thick HfO_2_ film is deposited using atomic layer deposition of TDMAH and H_2_O at 120 C, followed directly by 3 nm thick TiN deposition with TiCl_4_ and N_2_ at 120 C without breaking vacuum. The 150 nm thick Ag top electrode is then patterned and deposited using e-beam evaporation, followed by a blanket TiN isolation etch in CHF_3_ and electrical testing.

### Details of Onrstein-Uhlenbeck (OU) process

The OU process is a stochastic process which was introduced as a generalized Brownian motion model. When the velocity of a moving particle within a liquid or gas is modeled as a Brownian motion, the position of the particle at the next time step follows a Gaussian distribution with a zero mean and a variance *αt*, where *α* is a positive constant and *t* is time. However, the trajectories of the Brownian process are not differentiable, meaning that the time derivative does not exist, and the variation is unbounded in any finite time interval. The OU processes provide a way to overcome the problem and thus can be seen as a generalization of the standard Brownian motion model (Wiener process)^50^.

We calibrate the parameters of Eq. (5) using the experimentally measured threshold voltage *V*_*T*_ of 17 selector devices such as shown in Fig. 3f. We use the method of linear regression, which has been established in^51^ to recast the Eq. (5) to:

8 $$y={ax}+b+\epsilon$$

where *a* is the slope, *b* is the interception term and *ϵ* is a white noise term. The solution of Eq. (5) after discretization using the Euler-Maruyama method is given by:

9 $${{V}_{T}}_{t+1}={V}_{{T}_{t}}{e}^{-\theta \Delta t}+\mu \left(1-{e}^{-\theta \Delta t}\right)\sigma \sqrt{\frac{1-{e}^{-2\theta \Delta t}}{2\theta }}{{{{{\mathscr{N}}}}}}\left({{{{\mathrm{0,1}}}}}\right)$$

By comparing Eqs. (8) and (9), we have $a={e}^{-\theta \Delta t}$, $b=\mu \left(1-{e}^{-\theta \Delta t}\right)$ and sd$(\epsilon )=\sigma \sqrt{\frac{1-{e}^{-2\theta \Delta t}}{2\theta }}$. Solving for *a*, *b* and sd(*ϵ*), we obtain the OU parameters $\mu =\frac{b}{1-a},\,\theta =-\frac{{{{{{\rm{ln}}}}}}a}{\Delta t}$ and $\sigma ={{{{{{\rm{sd}}}}}}}(\epsilon )\sqrt{\frac{-2{{{{{\rm{ln}}}}}}a}{\Delta t(1-{a}^{2})}}$. We have to compute *a*, *b* and the variance of the error of the linear regression in order to calibrate the OU parameters *μ*, *θ* and *σ*. The least square regression terms are ${S}_{x}=\mathop{\sum }\limits_{i=1}^{n}{S}_{i-1},\,{S}_{y}=\mathop{\sum }\limits_{i=1}^{n}{S}_{i},\,{S}_{{xx}}=\mathop{\sum }\limits_{i=1}^{n}{S}_{i-1}^{2},\,{S}_{{xy}}=\mathop{\sum }\limits_{i=1}^{n}{S}_{i-1}{S}_{i}$ and ${S}_{{yy}}=\mathop{\sum }\limits_{i=1}^{n}{S}_{i}^{2}$ where *S* represents a sample drawn from the experimental data. Upon further simplification, we end up with computing the following equations:

10 $$a=\frac{n{S}_{{xy}}-{S}_{x}{S}_{y}}{n{S}_{{xx}}-{S}_{x}^{2}}$$

11 $$b=\frac{{S}_{y}-{{aS}}_{x}}{n}$$

12 $${{{{{{\rm{sd}}}}}}}(\epsilon )=\sqrt{\frac{n{S}_{{yy}}-{S}_{y}^{2}-a\left(n{S}_{{xy}}-{S}_{x}{S}_{y}\right)}{n(n-2)}}$$

The parameter *σ* is computer as the ratio of $\frac{{{{{{{\rm{sd}}}}}}}\left(\epsilon \right)}{\sqrt{\Delta t}}$, where *Δt* is the sampling step for the experimental data or the time step of the Euler-Maruyama method.

### Training process of NSM

The MLP network described in Fig. 4a was trained with the backpropagation algorithm^52^, the Cross-entropy as loss function and an adapted version of Adam optimizer with a learning rate of 0.0003 and betas (0.9, 0.999). We adapted the Adam optimizer to accommodate for the updates of the conductance in the FeFet model (see paragraph: Building Blocks for Stochastic Synapse: FeFET-based Analog Weight Cell). The training and testing batch sizes were both set to 100. We trained the network for 200 epochs and at each epoch we used the full 60,000 samples training MNIST set. The learning rate was linearly decreased after 100 epochs with a rate of $0.0003\times {\min }\left\{2-\frac{x}{100},1\right\}$, where *x* is the number of a specific epoch. Every two epochs we measured the accuracy of the network using the full 10,000 samples testing MNIST set over an ensemble of 100 samples of the forward pass of the neural network. The accuracy was measured as the ratio of successfully classified digits to the total number of samples within the test MNIST set (10,000). All the experiments ran on a Nvidia GPU Titan X with 12GB of physical memory and a host machine equipped with a Intel i9 with 64 GB physical memory running Arch Linux. The source code is written in Python (Pytorch, Numpy, Sklearn) and it will [be freely available online upon acceptance for publication].

## Data Availability

The data that support the findings of this study are available from the corresponding author upon request.

## Code availability

The simulation codes used for this study are available from the corresponding author upon request.

## References

[1] Yu, S. et al. Scaling-up resistive synaptic arrays for neuro-inspired architecture: challenges and prospect. In *Technical Digest*—*International Electron Devices Meeting, IEDM* (2015).

[2] Gao, L. et al. Fully parallel write/read in resistive synaptic array for accelerating on-chip learning. *Nanotechnology* **26**, 455204 (2015). 10.1088/0957-4484/26/45/455204 26491032

[3] Ambrogio, S. et al. Equivalent-accuracy accelerated neural-network training using analogue memory. *Nature* **558**, 60–67 (2018). 10.1038/s41586-018-0180-5 29875487

[4] Kuzum, D., Jeyasingh, R. G. D., Lee, B. & Wong, H. S. P. Nanoelectronic programmable synapses based on phase change materials for brain-inspired computing. *Nano Lett*. **12**, 2179–2186 (2012). 10.1021/nl201040y 21668029

[5] Burr, G. W. et al. Experimental demonstration and tolerancing of a large-scale neural network (165 000 synapses) using phase-change memory as the synaptic weight element. *IEEE Trans. Electron Devices* **62**, 3498–3507 (2015).

[6] Jerry, M. et al. Ferroelectric FET analog synapse for acceleration of deep neural network training. in *Technical Digest*—*International Electron Devices Meeting, IEDM* (2018).

[7] Sun, X., Wang, P., Ni, K., Datta, S. & Yu, S. Exploiting hybrid precision for training and inference: a 2T-1FeFET based analog synaptic weight cell. in *Technical Digest*—*International Electron Devices Meeting, IEDM* (2019).

[8] Luo, Y., Wang, P., Peng, X., Sun, X. & Yu, S. Benchmark of ferroelectric transistor based hybrid precision synapse for neural network accelerator. *IEEE J. Explor. Solid-State Comput. Devices Circuits* **5**, 142–150 (2019).

[9] Jerry, M. et al. *Ferroelectric FET based Non-Volatile Analog Synaptic Weight Cell* (University of Notre Dame, 2019).

[10] Dutta, S. et al. Supervised learning in all FeFET-based spiking neural network: opportunities and challenges. *Front. Neurosci*. **14**, 634 (2020). 10.3389/fnins.2020.00634 PMC7327100 32670012

[11] Tuma, T., Pantazi, A., Le Gallo, M., Sebastian, A. & Eleftheriou, E. Stochastic phase-change neurons. *Nat. Nanotechnol*. **11**, 693–699 (2016). 10.1038/nnano.2016.70 27183057

[12] Dutta, S. et al. Programmable coupled oscillators for synchronized locomotion. *Nat. Commun*. **10**, 3299 (2019). 10.1038/s41467-019-11198-6 PMC6656780 31341167

[13] Knill DC, Pouget A (2004). The Bayesian brain: the role of uncertainty in neural coding and computation. Trends Neurosci..

[14] Borst JGG (2010). The low synaptic release probability in vivo. Trends Neurosci..

[15] Neftci EO, Pedroni BU, Joshi S, Al-Shedivat M, Cauwenberghs G (2016). Stochastic synapses enable efficient brain-inspired learning machines. Front. Neurosci..

[16] Ackley, D. H., Hinton, G. E. & Sejnowski, T. J. A learning algorithm for boltzmann machines. *Cogn. Sci*. **9**, 147–169 (1985).

[17] Hinton, G. E., Srivastava, N., Krizhevsky, A., Sutskever, I. & Salakhutdinov, R. R. Improving neural networks by preventing co-adaptation of feature detectors. Preprint at 10.48550/arXiv.1207.0580 (2012).

[18] Wan, L., Zeiler, M., Zhang, S., LeCun, Y. & Fergus, R. Regularization of neural networks using DropConnect. In *30th International Conference on Machine Learning, ICML**2013* (2013).

[19] Buesing L, Bill J, Nessler B, Maass W (2011). Neural dynamics as sampling: a model for stochastic computation in recurrent networks of spiking neurons. PLoS Comput. Biol..

[20] Gal, Y. & Ghahramani, Z. Dropout as a Bayesian approximation: Representing model uncertainty in deep learning. In *33rd International Conference on Machine Learning, ICML 2016* (2016).

[21] Levy WB, Baxter RA (2002). Energy-efficient neuronal computation via quantal synaptic failures. J. Neurosci..

[22] Harris JJ, Jolivet R, Attwell D (2012). Synaptic energy use and supply. Neuron.

[23] Doya, K., Ishii, S., Pouget, A. & Rao, R. P. N. *Bayesian Brain: Probabilistic Approaches to Neural Coding* (MIT Press, 2007).

[24] Friston, K. The free-energy principle: a unified brain theory? *Nat. Rev. Neurosci*. **11**, 127–138 (2010). 10.1038/nrn2787 20068583

[25] Cohn DA, Ghahramani Z, Jordan MI (1996). Active learning with statistical models. J. Artif. Intell. Res..

[26] Yu S (2018). Neuro-inspired computing with emerging nonvolatile memorys. Proc. IEEE.

[27] Detorakis, G. et al. Inherent weight normalization in stochastic neural networks. In *Advances in Neural Information Processing Systems* 3286–3297 (2019).

[28] Salimans, T. & Kingma, D. P. Weight normalization: a simple reparameterization to accelerate training of deep neural networks. In *Advances in Neural Information Processing Systems* (2016).

[29] Ullmann M, Goebel H, Hoenigschmid H, Haneder T (2001). Disturb free programming scheme for single transistor ferroelectric memory arrays. Integr. Ferroelectr..

[30] Ni K, Li X, Smith JA, Jerry M, Datta S (2018). Write disturb in ferroelectric FETs and its implication for 1T-FeFET and memory arrays. IEEE Electron Device Lett..

[31] Jerry M (2018). A Ferroelectric field effect transistor based synaptic weight cell. J. Phys. D. Appl. Phys..

[32] Trentzsch, M. et al. A 28nm HKMG super low power embedded NVM technology based on ferroelectric FETs. In *Technical Digest*—*International Electron Devices Meeting, IEDM* (2017).

[33] Ni, K., Chakraborty, W., Smith, J., Grisafe, B. & Datta, S. Fundamental understanding and control of device-to-device variation in deeply scaled ferroelectric FETs. (2019).

[34] Shukla, N., Ghosh, R. K., Gnsafe, B. & Datta, S. Fundamental mechanism behind volatile and non-volatile switching in metallic conducting bridge RAM. In *Technical Digest*—*International Electron Devices Meeting, IEDM* (2018).

[35] Grisafe, B., Jerry, M., Smith, J. A. & Datta, S. Performance enhancement of Ag/HfO_2_ metal ion threshold switch cross-point selectors. *IEEE Electron Device Lett*. **40**, 1602–1605 (2019).

[36] Ioffe, S. & Szegedy, C. Batch normalization: accelerating deep network training by reducing internal covariate shift. In *32nd International Conference on Machine Learning, ICML**2015* (2015).

[37] Ren, M., Liao, R., Urtasun, R., Sinz, F. H. & Zemel, R. S. Normalizing the normalizers: comparing and extending network normalization schemes. In *5th International Conference on Learning Representations, ICLR 2017*—*Conference Track Proceedings* (2017).

[38] Querlioz D, Bichler O, Vincent AF, Gamrat C (2015). Bioinspired programming of memory devices for implementing an inference engine. Proc. IEEE.

[39] Kau, D. et al. A stackable cross point phase change memory. In *Technical Digest*—*International Electron Devices Meeting, IEDM* (2009).

[40] Shenoy RS (2014). MIEC (mixed-ionic-electronic-conduction)-based access devices for non-volatile crossbar memory arrays. Semiconductor Sci. Technol..

[41] Imada M, Fujimori A, Tokura Y (1998). Metal-insulator transitions. Rev. Mod. Phys..

[42] Berglund CN, Guggenheim HJ (1969). Electronic properties of VO_2_ near the semiconductor-metal transition. Phys. Rev..

[43] Wentzcovitch, R. M., Schulz, W. W. & Allen, P. B. VO_2_: Peierls or Mott-Hubbard? A view from band theory. *Phys. Rev. Lett*. **72**, 3389 (1994). 10.1103/PhysRevLett.72.3389 10056186

[44] Cha, E. et al. Comprehensive scaling study of NbO_2_ insulator-metal-transition selector for cross point array application. *Appl. Phys. Lett*. **108**, 153502 (2016).

[45] Kim, W. G. et al. NbO_2_-based low power and cost effective 1S1R switching for high density cross point ReRAM application. In *Digest of Technical Papers*—*Symposium on VLSI Technology* (2014).

[46] Midya, R. et al. Anatomy of Ag/Hafnia-based selectors with 10^10^ nonlinearity. *Adv. Mater*. **29**, 1604457 (2017). 10.1002/adma.201604457 28134458

[47] Li, Y. et al. High-uniformity threshold switching HfO_2_-based selectors with patterned Ag nanodots. *Adv. Sci*. **7**, 2002251 (2020). 10.1002/advs.202002251 PMC7675059 33240773

[48] Radu, I. P. et al. High performance oxide diode. In *Solid State Devices and Materials Conference-SSDM* 586–587 (2013).

[49] Bengio, Y., Léonard, N. & Courville. A. Estimating or propagating gradients through stochastic neurons for conditional computation. Preprint at 10.48550/arXiv.1308.3432 (2013).

[50] Kovalenko, I. N., Kuznetsov, N. Y. & Shurenkov, V. M. *Models of Random Processes: A Handbook for Mathematicians and Engineers* (CRC Press, 1996).

[51] Dixit, A. K. & Pindyck, R. S. *Investment Under Uncertainty, Princeton university press* (2012).

[52] Rumelhart DE, Hinton GE, Williams RJ (1986). Learning representations by back-propagating errors. Nature.

